# Evaluation of the Photocatalytic Properties of Copper Oxides/Graphene/TiO_2_ Nanoparticles Composites

**DOI:** 10.3390/molecules27185803

**Published:** 2022-09-07

**Authors:** Dragos Cosma, Alexandra Urda, Teodora Radu, Marcela C. Rosu, Maria Mihet, Crina Socaci

**Affiliations:** 1 National Institute for Research and Development of Isotopic and Molecular Technologies—INCDTIM, 67-103 Donat Street, 400293 Cluj-Napoca, Romania; 2 Faculty of Chemistry and Chemical Engineering, Babes-Bolyai University, 11 Arany Janos Street, 400028 Cluj-Napoca, Romania

**Keywords:** copper oxides-TiO_2_-graphene composite, methylene blue UVA photodegradation, Cu_2_O/CuO-TiO_2_ heterojunction

## Abstract

Easy and cost-efficient modifications of titanium dioxide nanoparticles that improve their efficiency in the visible light domain represent a continuous and challenging research topic. In addition, the effect of graphene on the overall photocatalytic process is still debated. Consequently, herein, we prepared a series of TiO_2_ nanoparticle-based composites with different copper oxide mass content (1–3%) and co-doped with graphene of different oxidation degrees. Different characterization techniques were used to analyze the structural and physico-chemical properties of the obtained composites: Scanning Electron Microscopy (SEM)/Transmission Electron Microscopy (TEM)/Energy-dispersive X-ray spectroscopy (EDX) analysis, X-ray powder diffraction (XRD), Fourier-transformed infrared spectroscopy (FT-IR) and X-ray photoelectron spectroscopy (XPS). The photocatalytic performance was evaluated by the degradation of methylene blue under both UVA and visible light irradiation. The nanocomposites show very good photocatalytic activity independent of the presence of reduced graphene oxide, due to the Cu_2_O/CuO-TiO_2_ heterojunctions. This finding has been confirmed by the very efficient visible-light-driven degradation of amoxicillin and ciprofloxacin.

## 1. Introduction

Our current society has a growing need for developing green and economical technologies that make the most out of sunlight. In this context, photodegradation of organic pollutants is of continuous interest to the scientific community, either for dyes [1] or emerging contaminants [2] removal. Titanium dioxide nanoparticles (TiO_2_) are widely investigated as catalysts in photo-driven processes, and details regarding their advantages and disadvantages have been largely described [3]. In order to benefit from their chemical stability, low price, non-toxicity, and debatable biocompatible nature, we should effectively use their semiconductor ability under solar irradiation. Because of this, researchers in the field have tested various approaches, and combined titania with different metals (alkaline, alkaline-earth, transition, and rare earth elements), or non-metals (including carbon-based nanomaterials) [4]. The main goal was the reduction in the band-gap, together with increasing the efficiency of charge separation by introducing species as electron traps. From the very large variety of titanium dioxide nanoparticle-doping possibilities, we are interested in plasmonic metals on one hand [5], and graphene-based nanomaterials, on the other hand [6]. Among the plasmonic metals, copper presents the best potential, either as reduced metal, or as an oxide (Cu_2_O and CuO). A cuprous oxide (Cu_2_O)/reduced graphene oxide composite proved to be an efficient visible-light photocatalyst for sulfamethoxazole removal [7]. Moreso, CuO promoted the enhancement of photocatalytic performance in a three-dimensionally ordered CuO-TiO_2_ composite [8]. Other studies have also stated the benefits of adding copper oxides to titania nanoparticles for photocatalytic and sensing applications [9,10,11,12]. Regarding the visible-light-induced photocatalytic activity for the degradation of methylene blue, mixed phase Cu_2_O/CuO nanorods have been studied and the authors reported on the importance and stability of both copper oxides, together [13]. A recent study explored the influence of the Cu_2_O ratio in CuO/Cu_2_O photocatalysts for methylene blue degradation under visible light and showed a more efficient process when the content of Cu_2_O is higher [14]. The incorporation of TiO_2_ in a CuO/Cu_2_O composite led to an increase in the bandgap energy. Even so, methylene blue photodegradation under visible light improved from about 80% to almost 100% [15]. In addition, the preparation of reduced graphene oxide with either CuO nanorods or Cu_2_O nanospheres by a simple method has been reported [16]. The authors concluded that reduced graphene oxide improves the visible-light-driven photocatalytic activity toward methylene blue.

Given the above, we considered it to be of interest to decorate the titania nanoparticles with copper oxides (Cu_2_O/CuO) nanoparticles on the surface (three different weight concentrations 1–3%), and also with graphene of different oxidation degrees—graphene oxide, thermally partially reduced graphene oxide at 200 °C, and thermally reduced graphene oxide at 300 °C. For this, we developed a preparation method that simultaneously accesses both copper oxides, leading to a composite of semiconductors (Cu_2_O/CuO-TiO_2_) with reduced graphene oxide. Even though composites containing both Cu_2_O and CuO onto reduced graphene oxide were reported efficient for photocatalysis [17], their preparation method is more tedious involving the use of chemical reagents for graphene oxide reduction purposes. Herein, we used a thermal reduction method, and a cleaner and cost-efficient process, with lower toxicity, which avoids the influence of other chemical elements traces. The evaluation of the influence of graphene oxidation degree on the properties of copper oxide containing titania has not been published so far. The structural and surface chemistry aspects of the materials were characterized by X-ray powder diffraction (XRD), FT-IR spectroscopy, and X-ray Photoelectron spectroscopy (XPS). The optical properties were assessed by diffuse reflectance (DRS) UV–Vis spectroscopy measurements.

The photocatalytic performance of the prepared catalysts was investigated by the degradation of methylene blue exposed to either ultraviolet light (UVA LED; centered at 350 nm), or visible light irradiation (white LED, 420–800 nm), in order to compare the activity of these catalysts with other reported photocatalysts. We started with the promising idea that the photodegradation of methylene blue by a film-deposited semiconductor develops into an ISO standard [18]. Moreover, for methylene blue, most of the papers above have reported a well-defined optical absorption and good resistance to light degradation, which now we agree only for the exposure to light of a certain intensity.

## 2. Results and Discussions

**Scheme 1:**
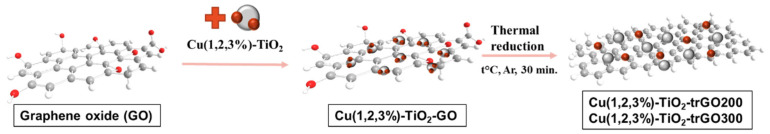
Preparation of Cu-TiO_2_-graphene composites.

The first step in the preparation of the investigated composites was the obtaining of the Cu(1, 2, or 3%)-TiO_2_ by the thermal decomposition of the impregnated copper nitrate, followed by reduction with a gaseous mixture of 10.5vol.% H_2_ in Ar (Scheme 1). The obtained titania nanoparticles are decorated with copper oxides (Cu_2_O-CuO), as detailed further in the XPS section. By mixing them with graphene oxide, we obtained the so-called Cu(1,2,3%)-TiO_2_-GO composites. Further thermal treatment, under argon, at either 200 or 300 °C led to Cu(1,2,3%)-TiO_2_-trGO200, or Cu(1,2,3%)-TiO_2_-trGO300. The detailed experimental procedures are found in the Materials and Methods section.

### 2.1. Morphological and Structural Characterization of the Composites

**Figure 1:**
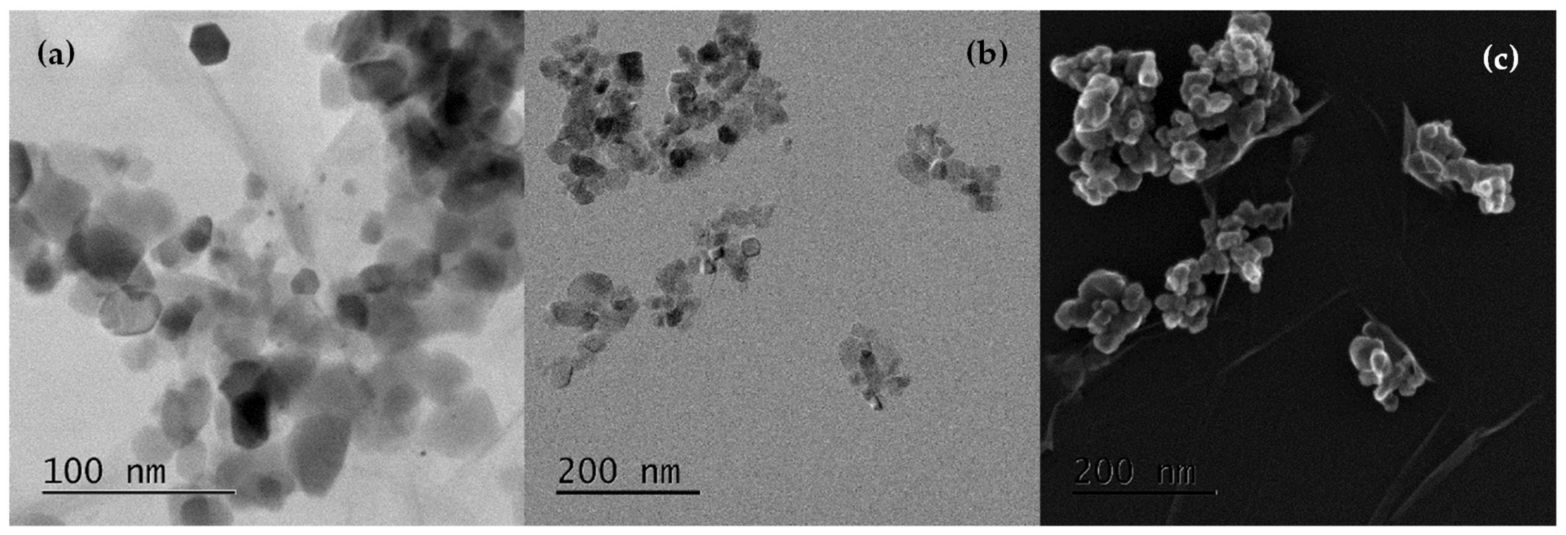
(**a**,**b**) TEM images of Cu(2%)-TiO_2_-trGO300; (**c**) SEM image of Cu(2%)-TiO_2_-trGO300.

The morphology of Cu(1,2,3%)-TiO_2_-graphene composites was investigated by TEM/SEM measurements, and is exemplified in Figure 1a–c for the composite with 2% copper content, and thermally reduced graphene oxide at 300 °C. The titania nanoparticles have a spherical shape and tend to agglomerate on the grid, with graphene being in close contact with them. The lower magnification image shows small copper oxide nanoparticles dispersed among the titania ones. As expected from the weight ratio of the three components in the composite, the titania nanoparticles dominate the suspended graphene nanosheets, which present out-of-plane corrugations, visible in the scanning mode (Figure 1c). The presence of copper species, together with carbon, oxygen, and titanium on the surface of the nanomaterial has been confirmed by the EDX spectrum (Figure S1—Supplemetary Materials).

The crystalline nature of the prepared composites was examined from the powder XRD spectra represented in Figure S2 (Supplemetary Materials), for the series with 3% copper content. As observed, all spectra have a similar pattern, the titania nanoparticles being crystallized in both anatase and rutile phases, with a clear exceeding of the anatase phase. The main characteristic diffraction peaks for TiO_2_ are present at 25.3°, 37.8°, 48.0°, 53.9°, 62.7°, and 75.0° 2θ angles, which were assigned to the respective (101), (004), (200), (105), (204), and (215) crystal planes of anatase phase TiO_2_ (JCPDS card file no. 21-1272). As previously reported [19], the diffraction lines belonging to the graphene and copper oxides were not detected due to the low amounts of these components. Their presence in the composite did not introduce any phase transformation of the titania nanoparticles.

The structural characterization was further deepened with the FT-IR spectra of the nanocomposites containing 3% copper (Figure S3, Supplemetary Materials). These spectra present the pattern of TiO_2_, showing the characteristic peaks at 3439 cm^−1^ for the stretching, 1525 cm^−1^ for the bending, and 1375 cm^−1^ for the deformation of the O–H bond. The intense peak at around 600 cm^−1^ is assigned to the Ti-O stretching band. In addition, the spectra of the composites with graphene show the C-O-C vibrations at 1065 cm^−1^ (only for graphene oxide), and 1138 cm^−1^. The characteristic bands of carbon–carbon bands are shielded by the TiO_2_ peaks.

The surface chemical composition of the composites was determined from XPS spectra (see Tables S1–S3 in the Supplemetary Materials). The XPS analysis of the prepared samples confirms the presence of Cu2p, O1s, and C1s peaks, indicating the successful preparation of the Cu_2_O/CuO-containing nanocomposites. The deconvolution of high-resolution XPS data was used to quantify the atomic percentage of each identified chemical bond.

**Figure 2:**
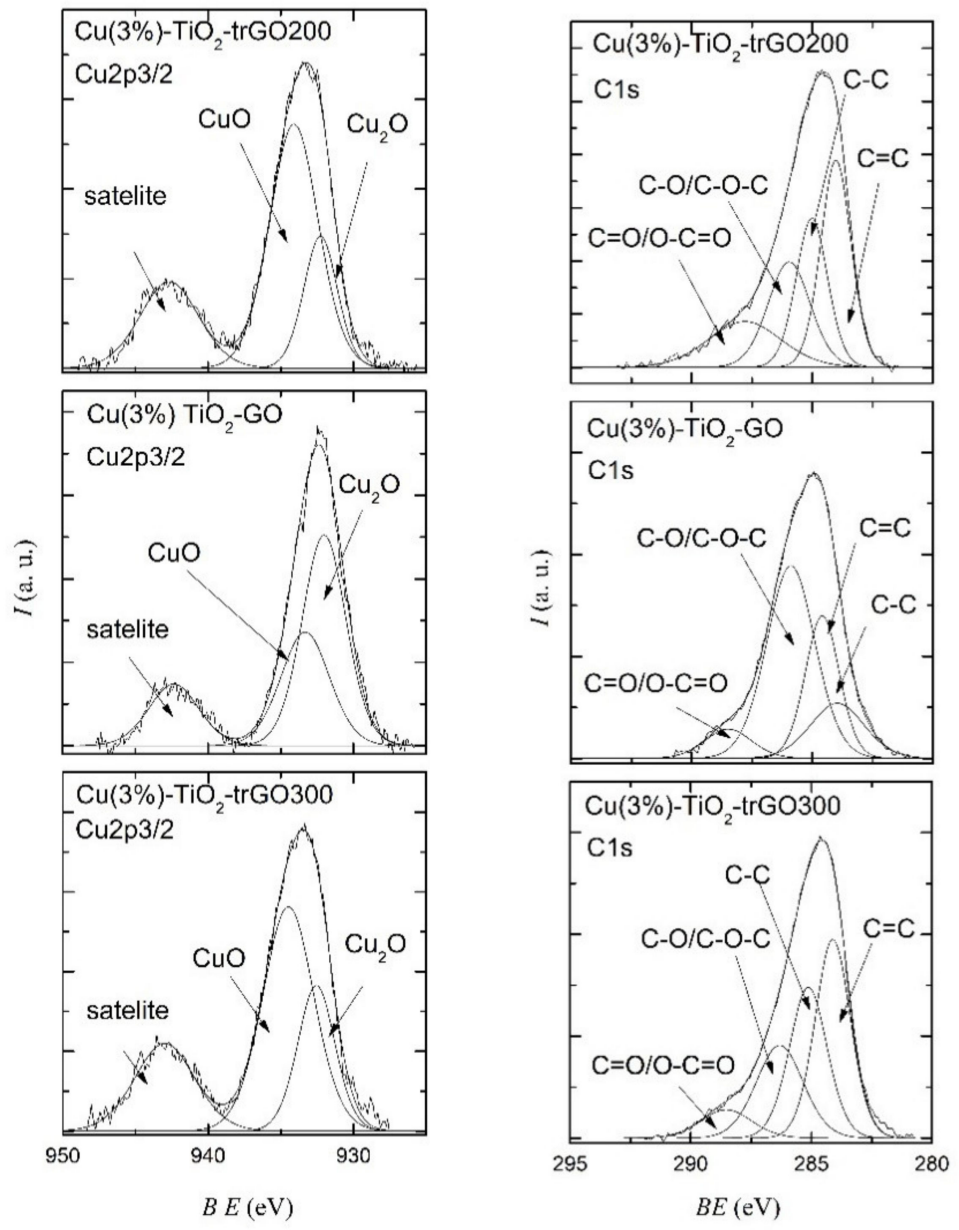
(**Left**) Cu2p high-resolution XPS spectra with the deconvolution components of the investigated samples; (**Right**) C1s XPS high-resolution spectra with the identified deconvolution components for the investigated samples.

The Cu2p energy level of all samples is composed of main characteristic doublet peaks corresponding to Cu2p1/2 and Cu2p3/2 at ~952.6 eV, and ~932.4 eV, respectively, and shake-up satellite structures at about 10 eV higher than that of the main Cu2p peak, and distinctly broader. The spin-orbit splitting between the two main photoemission peaks (Cu2p1/2 and Cu2p3/2) was determined to be around 20 eV, and was in good agreement with the values reported in the literature [20]. The satellite peak on the high binding energy region is known to be characteristic of the CuO phase of the core level Cu2p XPS data, and originates from multiple excitations in copper oxides. Therefore, the presence of the shake-up satellite structures observed in the Cu2p XPS spectra of the investigated samples was an indication of the presence of CuO species in the surface layer. The broad Cu2p3/2 peak and shake-up satellite feature were deconvoluted as shown in Figure 2, for each sample. For the sample Cu(3%)-TiO_2_-GO, the peaks located at 932 eV and 933.3 eV can be assigned also to Cu^+^ and Cu^2+^, respectively. The Cu2p shape line for the Cu(3%)-TiO_2_-trGO200 and Cu(3%)-TiO_2_-trGO300 samples are similar to previously investigated samples with the identified peaks related to CuO and Cu_2_O phases. This can be an important finding for the proposed applications of the prepared samples as proved by recent work [21], which showed that Cu_2_O/CuO heterojunction greatly accelerates the interface charge transfer of the heterojunction, enhancing the photoelectrochemical performance and the stability of the sample.

The C1s deconvolution was performed on each C1s spectrum to identify the chemical bonding of C atoms (Figure 2, right). In the case of each sample, four components were identified at the binding energies 284–284.3 eV, 284.6–285.1 eV, 285.4–286.3 eV, and 287–288.5 eV assigned to sp^2^ hybridized carbon atoms, sp^3^ hybridized carbon atoms, C–O/C–O–C of hydroxyl or epoxy group, C=O/O–C=O of carbonyl groups, carboxy acid, or ester groups, respectively [22].

The position of the Ti2p doublet for the investigated samples and the binding energy separation between the Ti2p3/2 and Ti2p1/2 peaks (5.7 and 5.8 eV), as shown in Figure S4a, indicate that, in all cases, the titanium was at the highest +4 oxidation state [23]. The normalized O1s spectra are shown in Figure S4b (Supplemetary Materials).

### 2.2. Optical Properties

**Figure 3:**
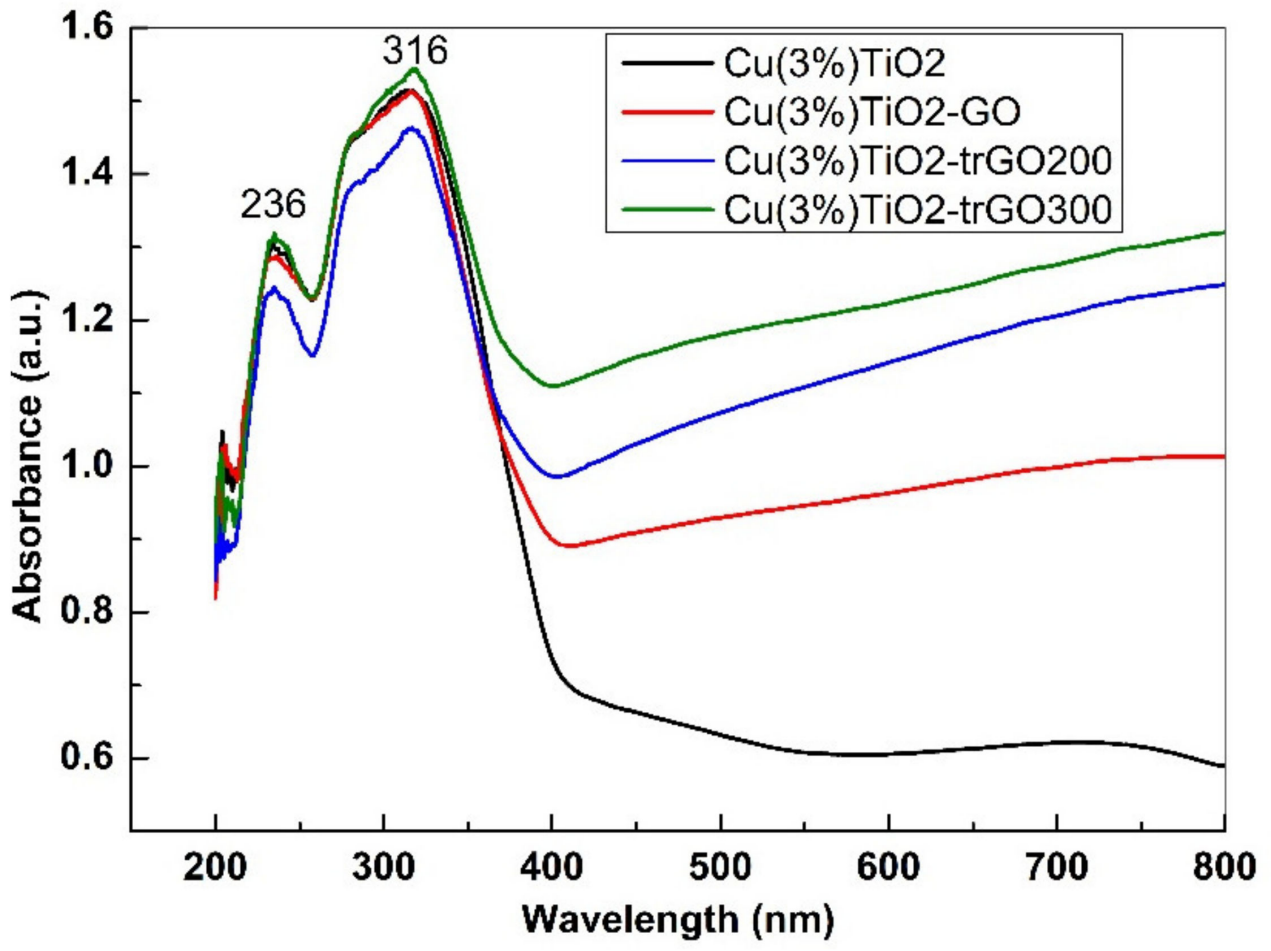
UV–Vis absorption spectra of Cu(3%)-TiO_2_-graphene composites.

The absorbance and diffuse reflectance UV–Vis spectra were measured in order to evaluate the optical characteristics of the composites, and the differences derived from the different reduction degrees of graphene oxide, or the copper content. The plotted spectra (Figure 3) show the characteristic absorbance peaks of titanium dioxide nanoparticles (236 nm and 316 nm). The Cu(3%)-TiO_2_ nanocomposite presents a broad absorbance around 700 nm, characteristic of CuO nanoparticles [24]. This band is covered by the graphene absorption band in the final composites. The values of absorption band gap energy (*E_g_*) of the photocatalysts (Table 1) were determined from the Tauc plots (Figure S5—Supplemetary Materials). The bandgap energies of the nanomaterials incorporating graphene are generally lower, decreasing with the reduction in functional groups on the graphene oxide surface. This clearly shows that reduced graphene oxide sheets have an impact on the optical properties of titanium dioxide nanoparticles. The copper content has only a small influence on the band gap energy, the lowest value is measured for the composites containing 2% copper.

### 2.3. Adsorption of Methylene Blue

**Figure 4:**
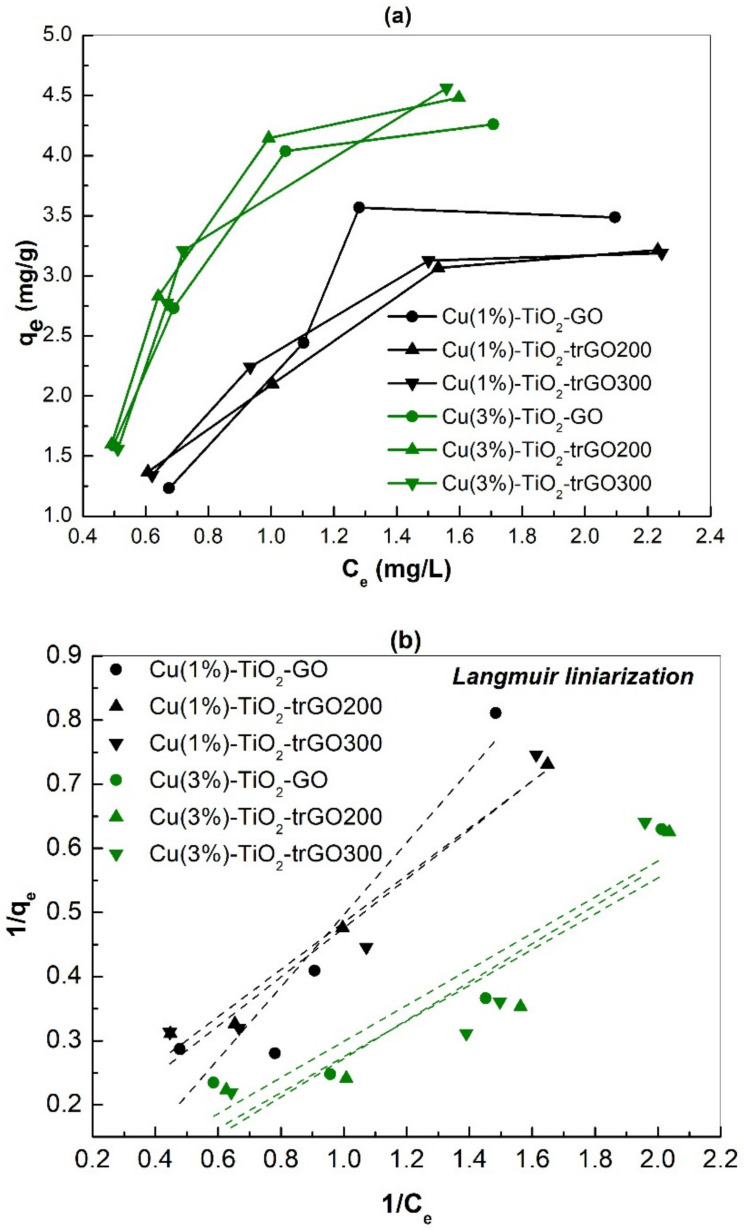

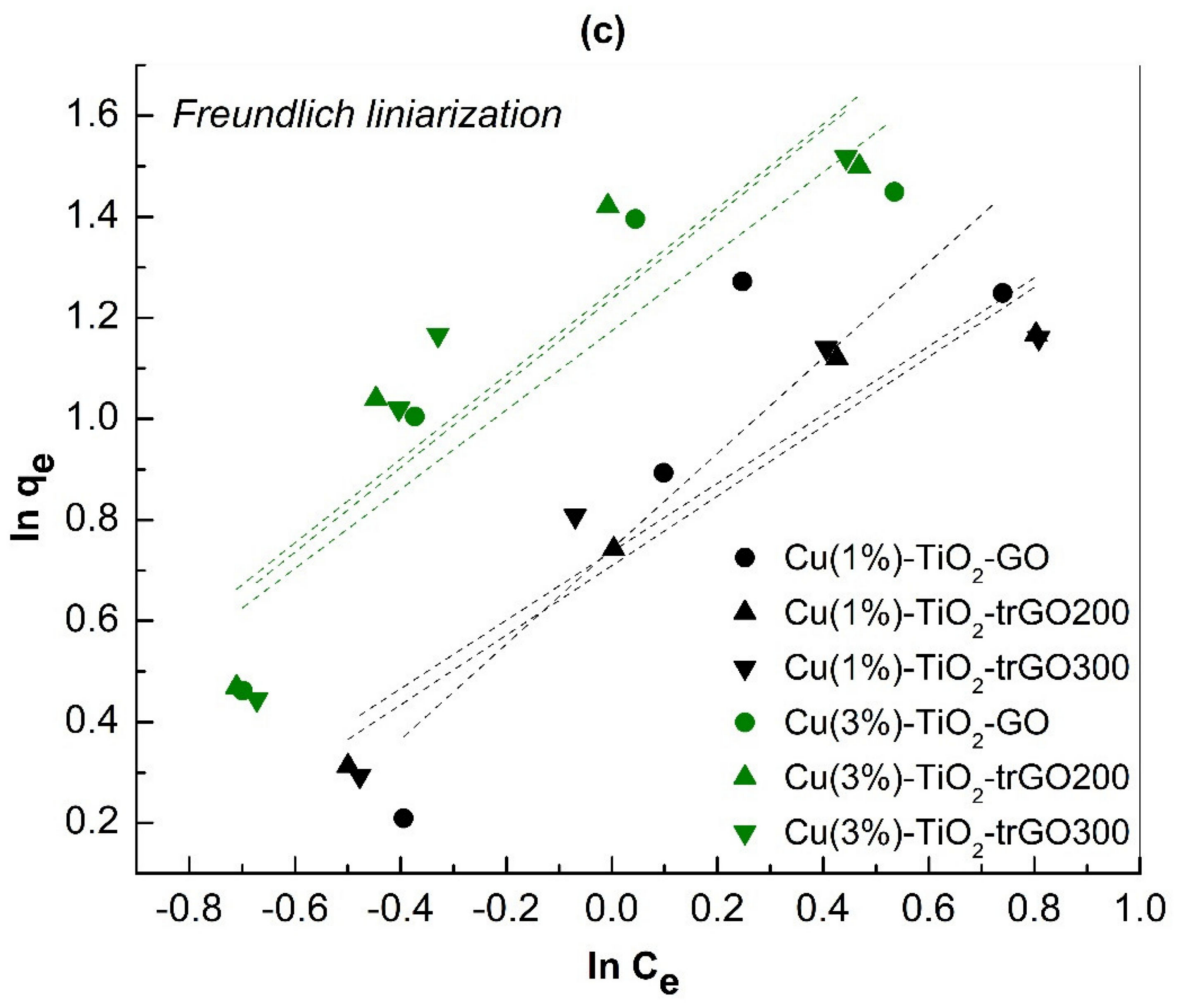
(**a**) Adsorption isotherms of MB dye onto Cu(1%)-TiO_2_ (black) and Cu(3%)-TiO_2_ (green)-graphene containing photocatalysts; (**b**) The Langmuir adsorption isotherm model fit for the adsorption of MB dye on Cu(1%) (black) and Cu(3%) (green)-graphene containing photocatalysts (experimental conditions: 120 min, 25 °C); (**c**) The Freundlich adsorption isotherm model fit for the adsorption of MB dye on Cu(1%) (black) and Cu(3%) (green)-graphene containing photocatalysts (experimental conditions: 120 min, 25 °C).

As previously stated, the adsorption and photodegradation processes were simultaneously studied. For the batch adsorption experiments, the photocatalyst concentration was kept constant, and the methylene blue concentration was varied over a 120 min period of time in order to reach the adsorption/desorption equilibrium. The isotherms for the adsorption process of methylene blue dye onto the copper-containing titania and graphene, at room temperature (25 °C), are shown in Figure 4a for the Cu(1 or 3%)-TiO_2_-graphene composites.

The quantitative evaluation of the equilibrium data of methylene blue adsorption on 1 or 3% copper-containing titanium dioxide nanoparticles and graphene composites was realized by the Langmuir and Freundlich adsorption isotherm models. Each of the two models offers some information on the adsorption process and mechanism. The linear fittings according to the Langmuir model are presented in Figure 4b. The extracted adsorption parameters (Table S4—Supplementary Materials) show that the Cu(1%) composites fit better than Cu(3%) in this model (with larger R^2^ values); the maximum adsorption capacity (*q_m_*) is 8–10 mg methylene blue/g photocatalyst. The R_L_ value is situated between 0 and 1 for the 1% copper-containing photocatalysts, indicating a favorable adsorption process. The linear fittings according to the Freundlich model are presented in Figure 4c, and the extracted parameters indicate a favorable adsorption process with physical interaction. The R^2^ values generally show a better fit to the Freundlich model for the 3% copper-containing materials.

### 2.4. Photodegradation Experiments

**Figure 5:**
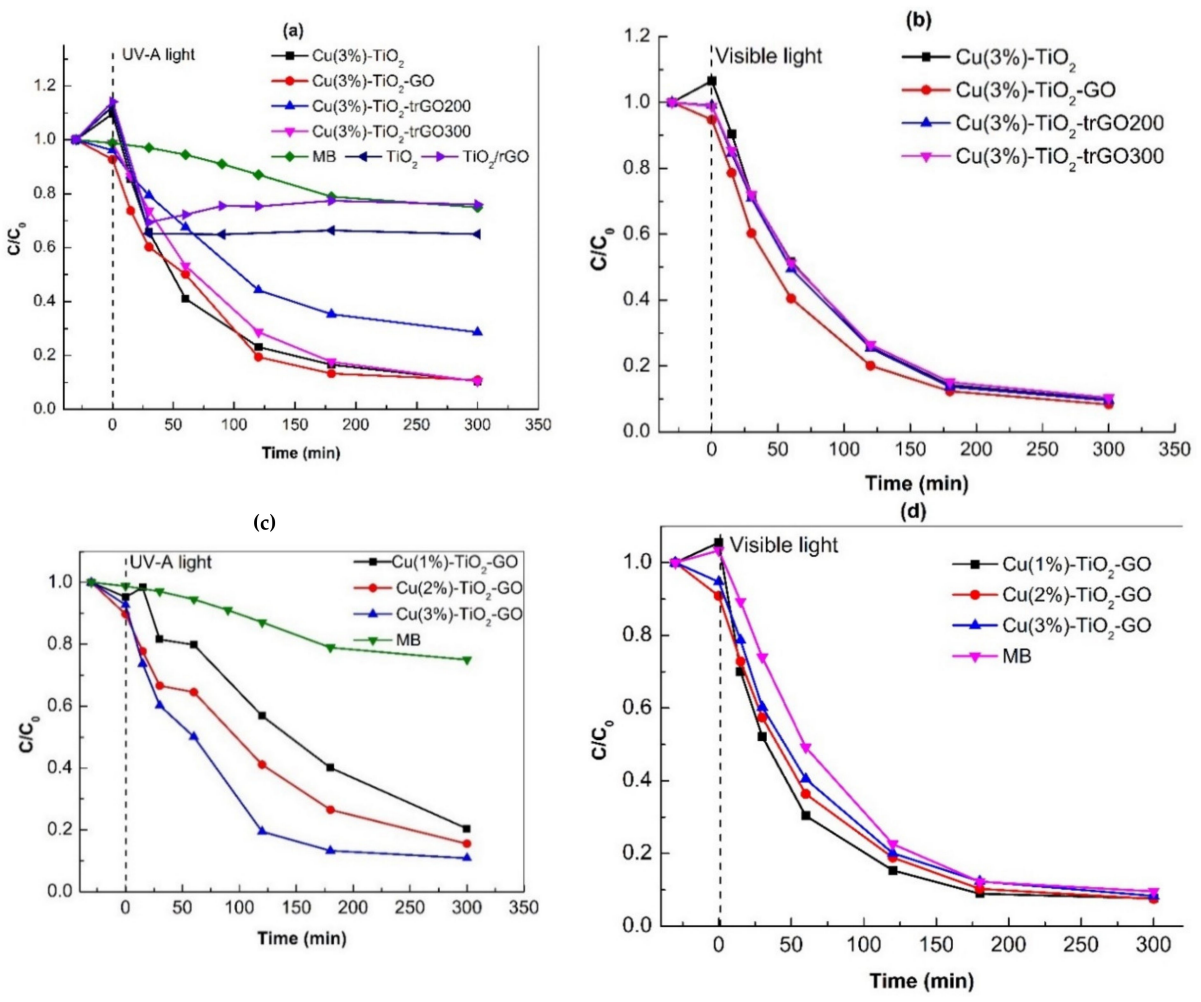
Residual ratio of MB (starting 5.5 mg/L) after UV-A irradiation (**a**,**c**), and visible light exposure (**b**,**d**), at a 0.5 mg/mL photocatalyst loading.

The decrease in methylene blue (5.5 mg/L; 1.7 × 10^−5^ M) solution concentrations *versus* time during the overall adsorption/photodecomposition process under visible and UV-A light, for Cu (1,2,3%)-TiO_2_-graphene (0.5 mg/mL) is shown in Figure 5a–d. The data show little difference in the activity of the twelve composites. In the case of visible light irradiation, we found that methylene blue concentration decreases over time even without any catalyst (magenta line—Figure 5d). This fact is due to the dye sensitization effect, as methylene blue absorbs light in the visible region. This phenomenon has been previously well studied [25].

In order to have a fair comparison with previous data and to understand our results, we extracted data in the table below (Table 2). They show quite different MB photodegradation efficiencies for the same (e.g., TiO_2_) or comparable photocatalysts. The comparison was limited by the different initial MB concentrations, but also the employed light source (even for a similar MB starting concentration). The power of an irradiation lamp energy consumption (Watt) does not show the amount of light it produces (lumens). As such, without measuring the irradiance it is hard to find common ground for comparing the many different photocatalysts [26]. We assume that MB self-degradation is dependent not only on the irradiation domain, but also on the light intensity.

The self-degradation of methylene blue under visible light irradiation leaves less room for the photodegradation process to be measured or compared with other composites (Figure 5b,d). Still, as we were really interested in comparing the composite activity, we switched to UV-A irradiation [36]. Figure 5a presents the residual concentration of methylene blue in the presence of the studied photocatalysts and is compared to the methylene blue alone (green line) and commercial TiO_2_ (dark blue line) and TiO_2_ with reduced graphene (violet line). The photodegradation experiments of methylene blue dye show the high efficiency of these catalysts, independent of the presence of any graphene (Figure 5a). This result suggests the prevalence of heterojunction Cu_2_O/CuO-TiO_2_ over the benefits of band-gap narrowing (Table 1 caused by the presence of graphene in the composites [12]. As a general observation, all the composites have a very good photocatalytic activity, with a slightly better performance than the Cu (3%)-containing ones (Figure 5c).

We calculated the apparent rate constant for a pseudo-first-order kinetic (*k_app_*) and the half-life time (t_1/2_) of the corresponding UV-A-driven photocatalytic processes (Table 3), by using the simplified equations [37]. The highest photodegradation rate was registered for the TiO_2_ composite containing 3% copper oxides and graphene oxide.

The photocatalyst dose (for Cu(1%)-TiO_2_-GO and Cu(1%)-TiO_2_-trGO300) shows little influence on the photocatalytic degradation of methylene blue under UV-A irradiation (Figure S6a—Supplemetary Materials). The methylene blue concentration decay is more abrupt for the 0.25 mg/mL (dotted lines) in comparison with the 0.5 mg/mL load, indicating a faster process when less catalyst is employed. As far as the methylene blue concentration is concerned, a lower concentration is bleached faster (Figure S6b—Supplemetary Materials). The pH influence has also been checked for one of the catalysts and it seems to be minor (Figure S7—Supplementary Materials).

**Figure 6:**
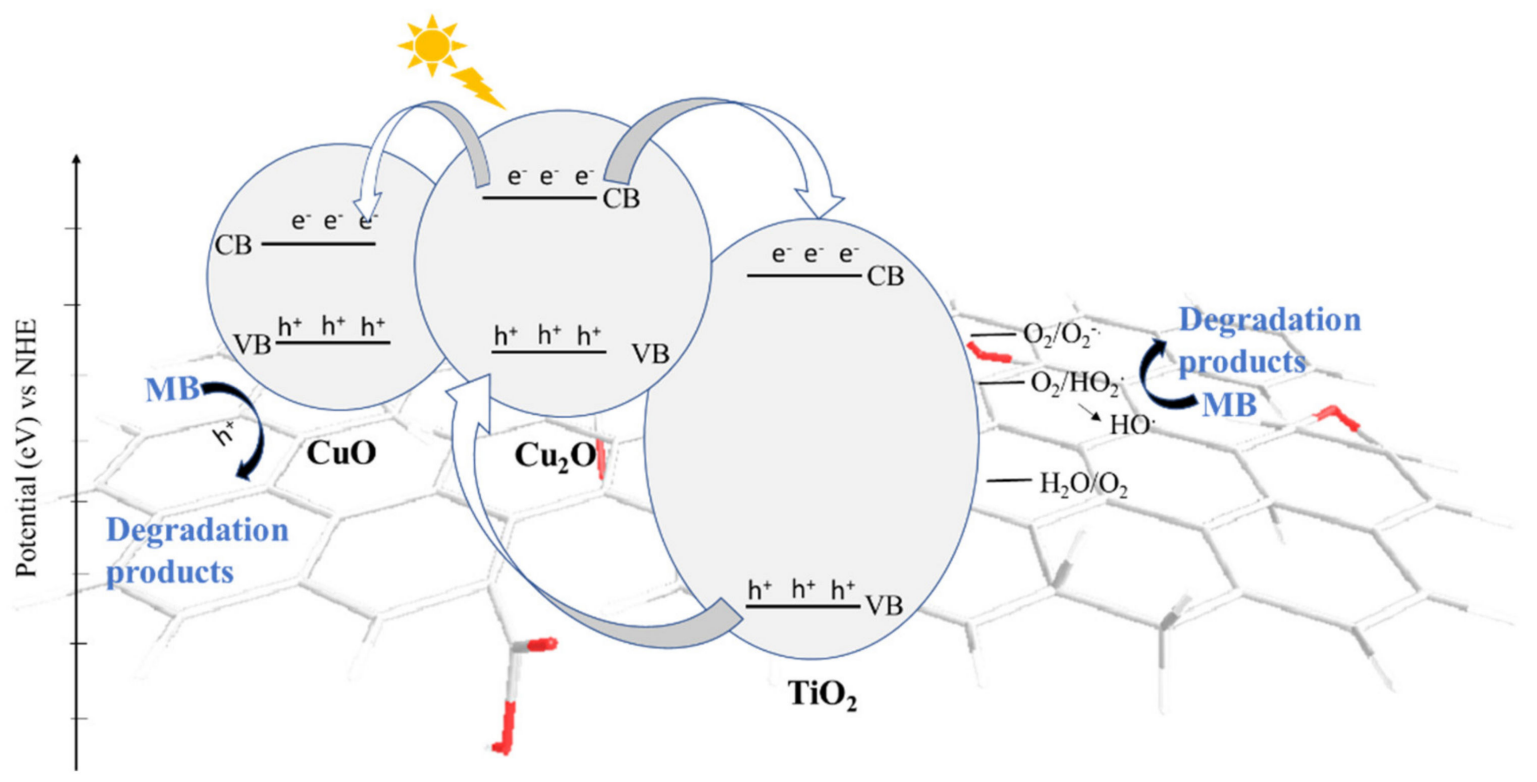
Schematic illustration for the Cu_2_O-CuO-TiO_2_ heterojunction.

As far as the photocatalytic mechanism is concerned, the literature states that our system is a type II heterojunction, with the photoexcited electrons from the conduction band of Cu_2_O being easily transferred to either the conduction band of TiO_2_ or CuO, due to the favorable positions of the bands (Figure 6). In this way, the holes migrate freely from one valance band to another, causing an efficient charge carrier separation even in the absence of graphene as an electron pool [12]. In addition, the mentioned conduction band positions are situated over the redox potential for the formation of reactive oxygen species that can decompose the methylene blue. The relative band positions of Cu_2_O-CuO and TiO_2_ relative to the redox potential for the formation of oxygen reactive species have previously been reported [38]. In addition, we cannot rule out a photosensitizer effect of the methylene blue absorbed at the surface of the nanocomposites [39]. In any case, the self-degradation of this dye hampers any clear conclusion.

**Figure 7:**
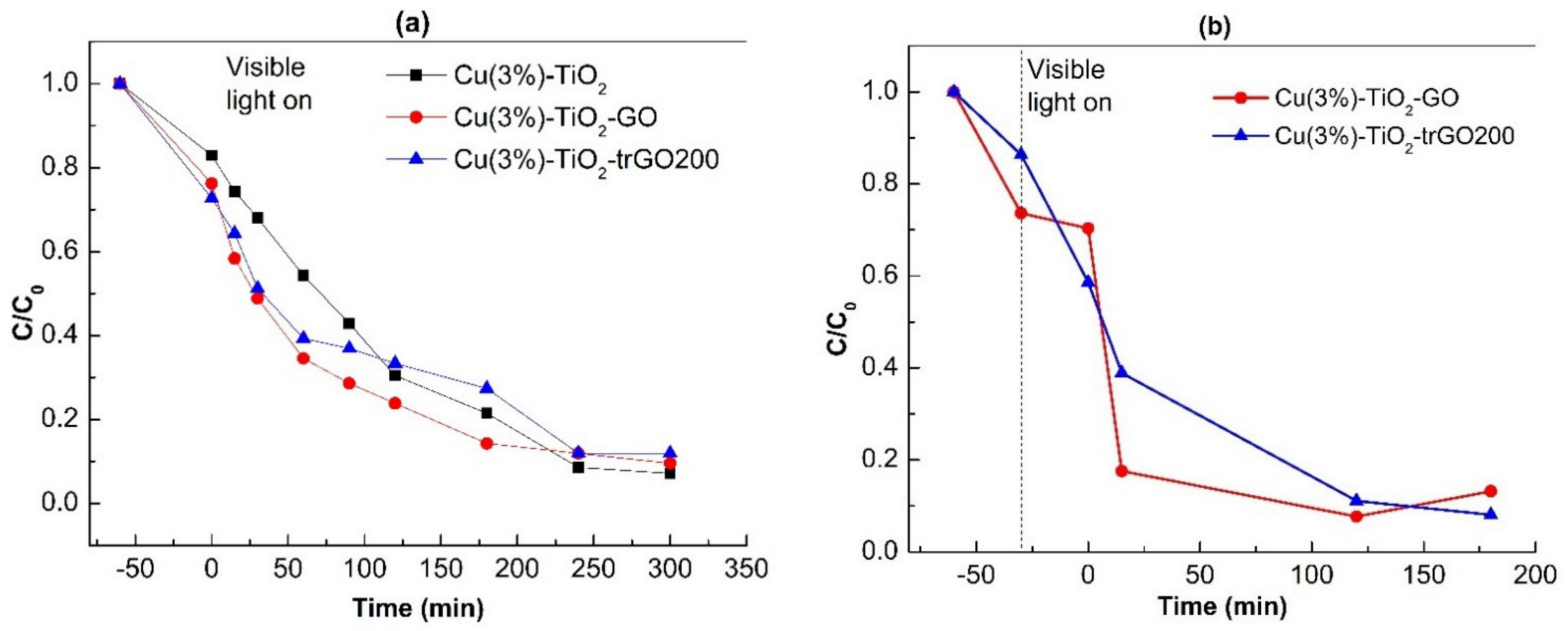
Residual ratio of (**a**) AMX (starting 1 mg/L) at a 0.5 mg/mL catalyst loading; (**b**) CPX (starting 2 mg/L) at a 0.2 mg/mL catalyst loading during adsorption/visible light degradation processes.

The results presented so far are definitely important, but our case study (methylene blue) has not been chosen properly due to misleading discoloration conclusions. In order to verify that the investigated composites (copper oxides and graphene containing titania nanoparticles) are good photocatalysts and to prove the importance of band edge positions of the heterojunction, we tested three of them for the visible-light-assisted photodegradation of amoxicillin (AMX) and ciprofloxacin (CPX). The two emerging organic pollutants are very well decomposed (Figure 7), independent of the reduction degree of the graphene oxide present in the composite.

## 3. Materials and Methods

All the reagents were commercially available and used as purchased. All water-based solutions were prepared using either distilled or Milli q water. Graphene oxide (GO) was prepared by a reported procedure [40]. Methylene blue (MB) and copper nitrate were purchased from Sigma-Aldrich; sodium phosphate, monobasic, anhydrous from Acros Organics; di-Sodium hydrogen phosphate anhydrous from VWR Chemicals, and titanium dioxide (TiO_2_-P25) from Evonik.

### 3.1. Preparation of Starting Cu(1,2,3%)-TiO_2_

The starting Cu-TiO_2_ nanocomposites were prepared by liquid impregnation method, according to the following procedure: Titanium dioxide (TiO_2_, 3 g) was impregnated with an appropriate aqueous solution of copper nitrate (Cu(NO_3_)_2_.3H_2_O) in order to obtain one of the targeted metal loadings (1, 2, or 3 wt%), and then was left to dry. The resulting powder was thermally treated at 450 °C, in an argon atmosphere for two hours, so that copper nitrate is decomposed to copper oxide nanoparticles. This was followed by a thermal treatment in a reducing gaseous mixture of 10.5 vol.% H_2_ in Ar, at a continuous flow and 280 °C for 90 min, in order to partially reduce the copper oxide to copper nanoparticles.

### 3.2. Preparation of Cu-TiO_2_-Graphene Composites

The freeze-dried GO (150 mg) together with 1.5 g of Cu(1,2,3%)-TiO_2_ was denoted as **Cu(1,2,3%)-TiO_2_-GO**. These composites were seated in a quartz boat into a temperature-programmed furnace. Argon gas was flown into the furnace chamber at a flow rate of 0.25 l min^−1^ and the temperature reached 300 °C in 30 min, resulting into Cu-TiO_2_ with thermally reduced graphene oxide at either 200 °C, denoted as **Cu(1,2,3%)-TiO_2_-trGO200,** or 300 °C, denoted as **Cu(1,2,3%)-TiO_2_-trGO300.**

### 3.3. Adsorption Isotherms/Photocatalytic Degradation of Methylene Blue (MB)

The adsorption and photodegradation steps were simultaneously studied, in the same experimental conditions, by changing the concentration of the methylene blue buffer solution. For this, prior to the irradiation, batch equilibrium experiments (adsorption/desorption) were performed in the dark for over 120 min. The effect of the initial concentration on its adsorption thermodynamics was determined using a given quantity of photocatalyst in phosphate buffer (pH = 7), and varying the MB concentration. After reaching equilibrium, the visible (or UVA) light was turned on, and, at various time intervals, the UV–Vis spectra of the solutions were measured. Finally, the results were calculated by the average values of triplicate measurements of triplicate experiments.

The consumed MB quantity/mass unit of photocatalyst (*q_e_*), and the residual ratio (C/C_0_) were calculated with the following formulae:

(1) $$q_{e}=\frac{\left(C_{\dot{i}}-C_{t}\right)V}{w}$$

(2) $$\frac{C}{C_{0}}=\frac{C_{t}}{C_{i}}$$

where, *C_i_* (µg/L) is the initial MB concentration, *C_t_* (µg/L) is the MB concentration at time *t*, *w* is the photocatalyst quantity (g), and *V* is the total solution volume (L).

### 3.4. Instrumental Part

The morphology of the composites was investigated by Scanning Transmission Electron Microscopy (STEM) at 200 kV using an H-7650 120 kV Automatic Microscope (Hitachi, Japan) and the SEM investigation was performed using an SU-8230 operated at 30 kV (Hitachi, Japan). The samples were prepared by dropping a few µL of diluted ethanol suspension on the nickel grid.

Fourier Transform Infrared (FT-IR) Spectra were recorded in a transmission mode on a Bruker Tensor II spectrometer (from KBr pellets) (Bruker Optics, Billerica, MA, USA). The UV–Vis absorption spectra were recorded on a V-570 JASCO Spectrophotometer (Jasco International Co, Ltd., Tokyo, Japan). X-Ray powder Diffraction (XRD) measurements were performed on a Bruker D8 Advance diffractometer (Billerica, MA, USA), using CuKα1 radiation (λ = 1.5406 Å).

X-ray photoemission spectroscopy (XPS) measurements were performed by using a PHOIBOS 150 2D CCD hemispherical energy analyzer XPS spectrometer (SPECS Surface Nano Analysis GmbH, Berlin, Germany) equipped with a multichanneltron detector, and an Al/Mg dual-anode as the excitation source. The vacuum in the measurement chamber was maintained at 1 × 10^−9^ Torr during the measurements. The XPS survey spectra were recorded at 30 eV pass energy, and 0.5 eV/step. The high-resolution spectra for the individual elements (Ag, C, O, Ti) were recorded by accumulating 10 scans at 30 eV pass energy and 0.1 eV/step. Data analysis and deconvolution of the spectra into the corresponding components were performed using Casa XPS software (version 2.3.24 PR1.0) with a Gaussian–Lorentzian (30) product function, and a nonlinear Shirley background subtraction. Peak shifts due to any apparent charging were normalized to the C1s peak set to 284.8 eV.

The photocatalytic degradation tests were carried out in a Luzchem LZC-4V (Ottawa, Ontario, Canada) Photoreactor equipped with 12 white LEDs (8 W) that emit in the visible domain 420–800 nm, the light intensity being 241.000 lx (lumen/m^2^). Comparison experiments with UVA LEDs (8 W, centered at 350 nm) with a light intensity of 885 lx (lumen/m^2^) were performed when considered necessary.

## 4. Conclusions

We developed an efficient, easy, and reproducible preparation method to decorate TiO_2_ nanoparticles with both Cu_2_O/CuO nanoparticles and graphenes with different reduction degrees. The spectroscopical and optical characterization of the composites showed a clear influence of the graphene oxidation/reduction degree on the band gap width values. Even though the beneficial presence of Cu_2_O/CuO heterojunction in the composites led to a very good photocatalytic performance, it seemed independent of the added graphene. This conclusion was based on the fact that titania nanoparticle-containing copper oxides proved to have similar photocatalytic efficiency towards methylene blue UV-A irradiation with the composites also containing graphene of different oxidation degrees. This behavior was confirmed in a test experiment using amoxicillin as an organic pollutant model. These results show the potential of copper oxides containing titania as efficient photocatalysts for water remediation under visible light exposure.

In addition, we confirmed that visible light exposure of methylene blue is influenced by its self-degradation. Different light sources (as far as the intensity and wavelength domains are concerned) influence the results of methylene blue photodegradation, limiting a direct comparison with other reported results.

## Figures and Tables

**Table 1:** Band gap (E_g_) calculated for the different composites using the Tauc method.

Copper Content (x%)	Cu(x%)-TiO_2_	Cu(x%)-TiO_2_-GO	Cu(x%)-TiO_2_-trGO200	Cu(x%)-TiO_2_-trGO300
**1%**	2.9 eV	2.75 eV	2.58 eV	2.5 eV
**2%**	2.94 eV	2.56 eV	2.34 eV	2.16 eV
**3%**	2.86 eV	2.76 eV	2.66 eV	2.56 eV

**Table 2:** Comparison of literature data on methylene blue photodegradation under different TiO_2_-containing catalysts and light exposure sources.

Light Source—Initial MB Concentration	Composite	MB Photodegradation	Ref
**UV** source—America brand lamp model F17T8/BLB 17W; (λmax = 360 nm)—2.3 × 10^−4^ M	TiO_2_	59%	[27]
	TiO_2_/graphene	87%	
**Visible** source—LED Philips 3PH5 with 14W-2.3 × 10^−4^ M	TiO_2_	-	
	TiO_2_/graphene	40%	
UV with λmax = 254 nm—1 × 10^−5^ M	TiO_2_/graphene (3wt%)	90%	[28]
**UV–Vis** light with high UV intensity—6 lamps with the power of 20W each, 110 W/m^2^ UV and 5 W/m^2^ Visible—10 mg/L	TiO_2_	29.42% (60 min)	[29]
	TiO_2_ 500	55% (60 min)	
	TiO_2_/rGO (1wt%)	63% (60 min)	
	TiO_2_/rGO (8wt%)	91.5% (60 min)	
**UV–Vis**—light source consisting of 2.31 µW/cm^2^ (220–280 nm), 6.94 mW/cm^2^ (315–400 nm), 129.3 mW/cm^2^ (400–1050 nm)—10 mg/L	MB alone	30% (50 min)	[30]
	TiO_2_ P25	100% (50 min)	
	Graphene-P25 3%	100% (80 min)	
	Graphene-TiO_2_-5%	100% (90 min)	
**Visible light**—84 mW/cm^2^ (400–1050 nm)—10 mg/L	Graphene-TiO_2_-3%	95% (150 min)	
**Visible light**—natural sunlight with a UV filter (11.45 a.m. to 17.45 p.m., 11.93oN; 79.13oE)—2 × 10^−5^ M	TiO_2_	40% (60 min)	[31]
	TiO_2_-graphene	80% (60 min)	
**UV** light—6 lamps of 20W each (310–430 nm), 110 W/cm^2^—10 mg/L	TiO_2_	38.78% (90 min)	[32]
	TiO_2_-700	86.48% (90 min)	
	TiO_2_/rGO-700	100% (90 min)	
**Visible** light—artificial solar light, halogen lamp, 60W—10 mg/L	TiO_2_	1.02% (300 min)	
	TiO_2_-700	7.11% (300 min)	
	TiO_2_/rGO-700	32.13% (300 min)	
**UV** light—H2100CH—5 lamps (λmax = 254 nm)—20 mg/L	TiO_2_/ZnO/rGO	99.6% (120 min)	[33]
**Visible light**—simulated solar light, Xenon lamp, 300W—20 mg/L	TiO_2_/ZnO/rGO	80% (180 min)	
**Visible** light—Xenon lamp, 500W—30 mg/L	TiO_2_-P25	2% (480 min)	[34]
	CuO/TiO_2_-GR	80% (480 min)	
**Visible** light—a Xenon lamp	Cu_2_O/TiO_2_	93.63% (45 min)	[35]
**Visible** light—150W lamp (OSRAM) with a 420 nm cutoff filter—5 mg/L	CuO-Cu_2_O/TiO_2_	90% (180 min)	[15]
**Solar** light—1 × 10^−5^ M	CuO	35% (60 min)	[16]
	CuO-rGO	50% (60 min)	
	Cu_2_O	45% (60 min)	
	Cu_2_O-rGO	52% (60 min)	
**UV** source—300W light source mainly UVA (315–400 nm) with some UVB (280–315 nm)—10 mg/L	Cu-doped TiO_2_/RGO (5 wt%)	64%	[19]
**Visible** light—Xenon lamp 300 W with a 420 nm cutoff filter—5 mg/L	Cu_2_O/rGO	100%	[7]

**Table 3:** The kinetic parameters corresponding to the most efficient photocatalysts for MB photodegradation.

	Cu(3%)-TiO_2_	Cu(3%)-TiO_2_-GO	Cu(3%)-TiO_2_-trGO200	Cu(3%)-TiO_2_-trGO300	Cu(1%)-TiO_2_-GO	Cu(2%)-TiO_2_-GO
*k_app_* **×** **10^3^ (min^−1^)**	10.10	11.40	5.85	10.10	4.91	6.15
**t_1/2_ (min)**	68.6	60.8	118.4	68.6	141.1	112.7
**R^2^**	0.9826	0.9885	0.9942	0.9990	0.9916	0.9914

## Data Availability

Not applicable.

## Supplementary Materials

The following supporting information can be downloaded at: https://www.mdpi.com/article/10.3390/molecules27185803/s1, Figure S1. Element mapping images of Cu(2%)-TiO_2_-trGO300 revealing the distribution of C (red), O (green), Ti (cyan), and Cu (magenta) elements and the corresponding EDX spectrum; Figure S2. The XRD patterns of Cu(3%)-TiO_2_-graphene composites; Figure S3. The FT-IR spectra of Cu(3%)-TiO_2_-graphene composites comparatively with Cu(3%)-TiO_2_; Figure S4. (a). Ti2p XPS for the investigated samples. Dashed line marks the spin orbit splitting of the Ti2p3/2 and Ti2p1/2 components. (b) O1s high-resolution spectra for the investigated samples. Inset shows the deconvolution of O1s spectra for Cu(3%)-TiO_2_-GO sample, as an exemplification of the identified chemical bonds at the surface of the sample. Figure S5. Tauc plots of all the analyzed samples (with 1%, 2%, or 3% copper content). Figure S6. Residual ratio of MB (starting 5.5 mg/L) after UV-A irradiation (at 0.5 and 0.25 mg/mL photocatalyst dose) (a); the starting MB concentration of 3.8 and 1.9 mg/L (b); Figure S7. The pH influence over the residual ratio of MB (starting 5.5 mg/L) after UV-A irradiation in the presence of Cu(1%)-TiO2-trGO300 photocatalyst; Figure S8. Recyclability study for Cu(1%)-TiO_2_-trGO300, 5.5 mg/L MB solution pH = 7 (30 min dark and 1 h irradiation with UVA light); Table S1. Elemental concentration at the surface of the investigated samples determined from XPS survey spectra of the analyzed samples; Table S2: Deconvolution components with the corresponding ratio determined from high-resolution Cu2p spectra of the analyzed samples; Table S3: Data obtained from the deconvolution of the C1s peaks of the investigated samples; Table S4. The adsorption isotherm models (Langmuir, Freundlich, and Temkin) parameters for MB on the studied photocatalysts.

## References

[1] Ruiz-Santoyo V., Andrade-Espinoza B.A., Romero-Toledo R., Anaya-Esparza L.M., Villagrán Z., Guerra-Contreras A. (2022). Use of Nanostructured Photocatalysts for Dye Degradation: A Reviewtle. Period. Polytech. Chem. Eng..

[2] Prakruthi K., Ujwal M.P., Yashas S.R., Mahesh B., Kumara Swamy N., Shivaraju H.P. (2022). Recent advances in photocatalytic remediation of emerging organic pollutants using semiconducting metal oxides: An overview. Environ. Sci. Pollut. Res..

[3] Zu M., Zhou X., Zhang S., Qian S., Li D.S., Liu X., Zhang S. (2021). Sustainable engineering of TiO_2_-based advanced oxidation technologies: From photocatalyst to application devices. J. Mater. Sci. Technol..

[4] Parangi T., Mishra M.K. (2019). Titania Nanoparticles as Modified Photocatalysts: A Review on Design and Development. Comments Inorg. Chem..

[5] Kumar A., Choudhary P., Kumar A., Camargo P.H.C., Krishnan V. (2022). Recent Advances in Plasmonic Photocatalysis Based on TiO_2_ and Noble Metal Nanoparticles for Energy Conversion, Environmental Remediation, and Organic Synthesis. Small.

[6] Karim A.V., Selvaraj A. (2021). Graphene composites in photocatalytic oxidation of aqueous organic contaminants–A state of art. Process Saf. Environ. Prot..

[7] Liu S.-H., Wei Y.-S., Lu J.-S. (2016). Visible-light-driven photodegradation of sulfamethoxazole and methylene blue by Cu_2_O/rGO photocatalysts. Chemosphere.

[8] Yang R., Yang L., Tao T., Ma F., Xu M., Zhang Z. (2014). Contrastive study of structure and photocatalytic performance with three-dimensionally ordered macroporous CuO–TiO_2_ and CuO/TiO_2_. Appl. Surf. Sci..

[9] Zhu H., Li Y., Jiang X. (2019). Room-temperature synthesis of cuprous oxide and its heterogeneous nanostructures for photocatalytic applications. J. Alloys Compd..

[10] Polat K. (2020). Cuprous oxide film sputtered on monolayer graphene for visible light sensitive heterogeneous photocatalysis. Thin Solid Films..

[11] Sahu K., Dhonde M., Murty V.V.S. (2021). Preparation of copper/TiO_2_/graphene oxide ternary nanocomposites and their structural, surface morphology, and optical properties. J. Mater. Sci. Mater. Electron..

[12] Janczarek M., Kowalska E. (2017). On the Origin of Enhanced Photocatalytic Activity of Copper-Modified Titania in the Oxidative Reaction Systems. Catalysts.

[13] Basnet P., Zhao Y. (2016). Tuning the CuxO nanorod composition for efficient visible light induced photocatalysis. Catal. Sci. Technol..

[14] Bayat F., Sheibani S. (2022). Enhancement of photocatalytic activity of CuO-Cu_2_O heterostructures through the controlled content of Cu_2_O. Mater. Res. Bull..

[15] Sabzehei K., Hadavi S.H., Bajestani M.G., Sheibani S. (2020). Comparative evaluation of copper oxide nano-photocatalyst characteristics by formation of composite with TiO_2_ and ZnO. Solid State Sci..

[16] Kumar S., Ojha A.K., Bhorolua D., Das J., Kumar A., Hazarika A. (2019). Facile synthesis of CuO nanowires and Cu_2_O nanospheres grown on rGO surface and exploiting its photocatalytic, antibacterial and supercapacitive properties. Phys. B Condens. Matter..

[17] Kottappara R., Palantavida S., Pillai S.C., Vijayan B.K. (2021). Composition tuning in copper - oxide decorated reduced graphene oxide yields efficient photo- and reduction catalysts. Surf. Interfaces.

[18] Mills A., Hill C., Robertson P.K.J. (2012). Overview of the current ISO tests for photocatalytic materials. J. Photochem. Photobiol. A Chem..

[19] Pham T.-T., Nguyen-Huy C., Lee H.-J., Nguyen-Phan T.-D., Son T.H., Kim C.-K., Shin E.W. (2015). Cu-doped TiO_2_/reduced graphene oxide thin-film photocatalysts: Effect of Cu content upon methylene blue removal in water. Ceram. Int..

[20] Akgul F.A., Akgul G., Yildirim N., Unalan H.E., Turan R. (2014). Influence of thermal annealing on microstructural, morphological, optical properties and surface electronic structure of copper oxide thin films. Mater. Chem. Phys..

[21] Wang P., Liu Z., Han C., Ma X., Tong Z., Tan B. (2021). Cu_2_O/CuO heterojunction formed by thermal oxidation and decorated with Pt co-catalyst as an efficient photocathode for photoelectrochemical water splitting. J. Nanopart. Res..

[22] Shen L., Zhang L., Wang K., Miao L., Lan Q., Jiang K., Lu H., Li M., Li Y., Shen B. (2018). Analysis of oxidation degree of graphite oxide and chemical structure of corresponding reduced graphite oxide by selecting different-sized original graphite. RSC Adv..

[23] Biesinger M.C., Payne B.P., Grosvenor A.P., Lau L.W.M., Gerson A.R., Smart R.S.C. (2011). Resolving surface chemical states in XPS analysis of first row transition metals, oxides and hydroxides: Cr, Mn, Fe, Co and Ni. Appl. Surf. Sci..

[24] Sawant S.S., Bhagwat A.D., Mahajan C.M. (2016). Synthesis of Cuprous Oxide (Cu_2_O) Nanoparticles—A Review. J. Nano-Electron. Phys..

[25] Sáenz-Trevizo A., Pizá-Ruiz P., Chávez-Flores D., Ogaz-Parada J., Amézaga-Madrid P., Vega-Ríos A., Miki-Yoshida M. (2019). On the Discoloration of Methylene Blue by Visible Light. J. Fluoresc..

[26] Bonfield H.E., Knauber T., Lévesque F., Moschetta E.G., Susanne F., Edwards L.J. (2020). Photons as a 21st century reagent. Nat. Commun..

[27] Acosta-Esparza M.A., Rivera L.P., Pérez-Centeno A., Zamudio-Ojeda A., González D.R., Chávez-Chávez A., Santana-Aranda M.A., Santos-Cruz J., Quiñones-Galván J.G. (2020). UV and Visible light photodegradation of methylene blue with graphene decorated titanium dioxide. Mater. Res. Express..

[28] Zhou F., Fu Y.Q., Wan X. (2012). Photocatalytic Enhancement in Methylene Blue Degradation of TiO_2_ Photocatalysts via Graphene Hybridization. Key Eng. Mater..

[29] Kusiak-Nejman E., Wanag A., Kapica-Kozar J., Kowalczyk Ł., Zgrzebnicki M., Tryba B., Przepiórski J., Morawski A.W. (2020). Methylene blue decomposition on TiO_2_/reduced graphene oxide hybrid photocatalysts obtained by a two-step hydrothermal and calcination synthesis. Catal. Today.

[30] Liu S., Sun H., Liu S., Wang S. (2013). Graphene facilitated visible light photodegradation of methylene blue over titanium dioxide photocatalysts. Chem. Eng. J..

[31] Alamelu K., Raja V., Shiamala L., Jaffar Ali B.M. (2018). Biphasic TiO_2_ nanoparticles decorated graphene nanosheets for visible light driven photocatalytic degradation of organic dyes. Appl. Surf. Sci..

[32] Wanag A., Kusiak-Nejman E., Czyżewski A., Moszyński D., Morawski A.W. (2021). Influence of rGO and Preparation Method on the Physicochemical and Photocatalytic Properties of TiO_2_/Reduced Graphene Oxide Photocatalysts. Catalysts.

[33] Nguyen C.H., Tran M.L., Van Tran T.T., Juang R.-S. (2020). Enhanced removal of various dyes from aqueous solutions by UV and simulated solar photocatalysis over TiO2/ZnO/rGO composites. Sep. Purif. Technol..

[34] Fang Y., Wang R., Jiang G., Jin H.E., Wang Y.I.N., Sun X., Wang S., Wang T.A.O. (2012). CuO/TiO_2_ nanocrystals grown on graphene as visible-light responsive photocatalytic hybrid materials. Bull. Mater. Sci..

[35] Ding R.C., Fan Y.Z., Wang G.S. (2018). High Efficient Cu_2_O/TiO_2_ Nanocomposite Photocatalyst to Degrade Organic Polluant under Visible Light Irradiation. ChemistrySelect.

[36] Mills A. (2012). An overview of the methylene blue ISO test for assessing the activities of photocatalytic films. Appl. Catal. B Environ..

[37] Lopes D., Daniel-da-Silva A.L., Sarabando A.R., Arias-Serrano B.I., Rodríguez-Aguado E., Rodríguez-Castellón E., Trindade T., Frade J.R., Kovalevsky A.V. (2020). Design of Multifunctional Titania-Based Photocatalysts by Controlled Redox Reactions. Materials.

[38] Luna A.L., Valenzuela M.A., Colbeau-Justin C., Vázquez P., Rodriguez J.L., Avendaño J.R., Alfaro S., Tirado S., Garduño A., De la Rosa J.M. (2016). Photocatalytic degradation of gallic acid over CuO–TiO_2_ composites under UV/Vis LEDs irradiation. Appl. Catal. A Gen..

[39] Vu Nu T.T., Thi Tran N.H., Truong P.L., Phan B.T., Nguyen Dinh M.T., Dinh V.-P., Phan T.S., Go S., Chang M., Loan Trinh K.T. (2022). Green synthesis of microalgae-based carbon dots for decoration of TiO_2_ nanoparticles in enhancement of organic dye photodegradation. Environ. Res..

[40] Pogacean F., Socaci C., Pruneanu S., Biris A.R., Coros M., Magerusan L., Katona G., Turcu R., Borodi G. (2015). Graphene based nanomaterials as chemical sensors for hydrogen peroxide—A comparison study of their intrinsic peroxidase catalytic behavior. Sens. Actuators B Chem..

