# Supplementary material for: Evaluation of the Photocatalytic Properties of Copper Oxides/Graphene/TiO2 Nanoparticles Composites
Source: Molecules. 2022 Sep 7;27(18):5803. doi: 10.3390/molecules27185803 (PMC9502858; doi:10.3390/molecules27185803)
Supplement: Supplementary file 1 [file molecules-27-05803-s001.zip › molecules-1877504-supplementary.pdf]

Supplementary Material

Evaluation of the photocatalytic properties of copper oxides/graphene/TiO_2 nanoparticles composites

Dragos Cosma[1], Alexandra Urda[1,2], Teodora Radu[1], Marcela C. Rosu[1], Maria Mihet[1], Crina Socaci*[1]

[1]National Institute for Research and Development of Isotopic and Molecular Technologies – INCDTIM, 67-103 Donat Street, 400293, Cluj-Napoca, Romania
[2]Faculty of Chemistry and Chemical Engineering, Babes-Bolyai University, 11 Arany Janos Street, 400028, Cluj-Napoca, Romania
*Correspondence: crina.socaci@itim-cj.ro

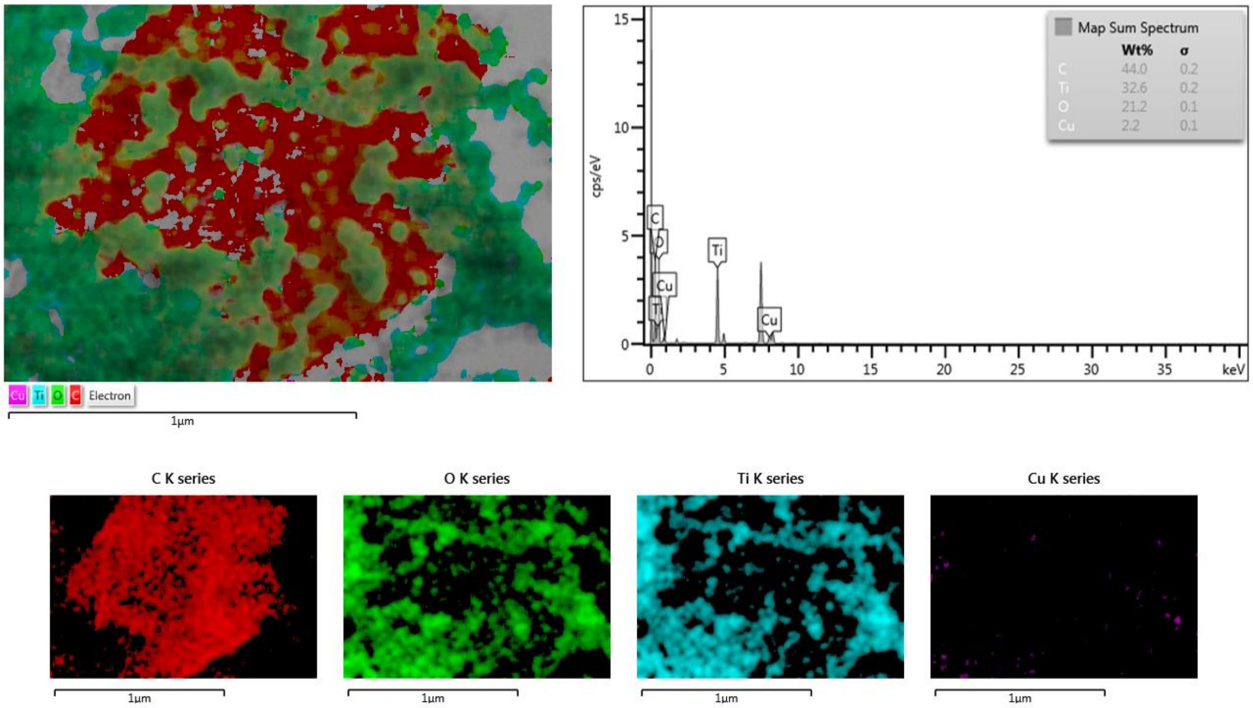

Figure S1. Element mapping images of Cu(2%)-TiO_2-trGO300 revealing the distribution of C (red), O (green), Ti (cyan) and Cu (magenta) elements and the corresponding EDX spectrum.

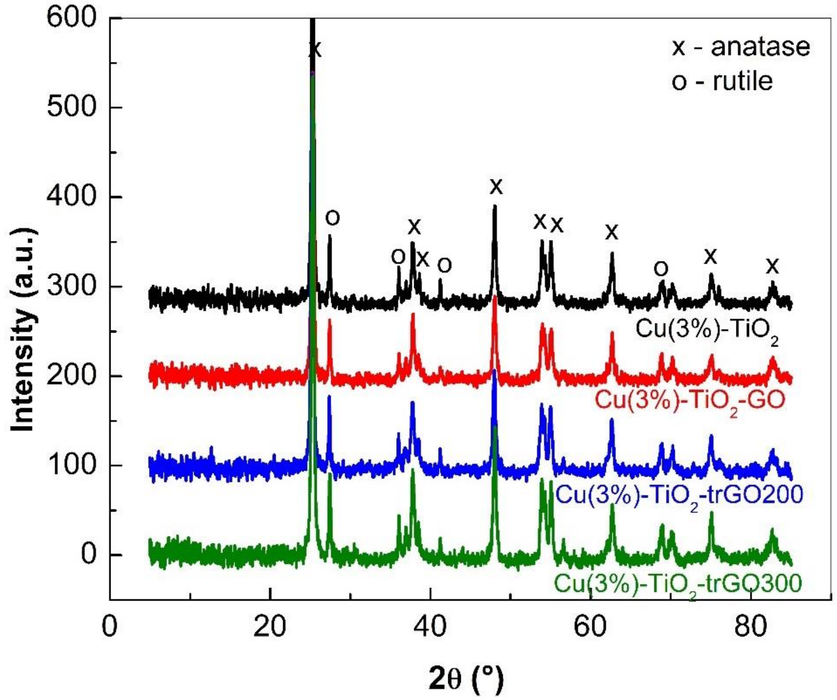

Figure S2. The XRD patterns of Cu(3%)-TiO_2-graphene composites.

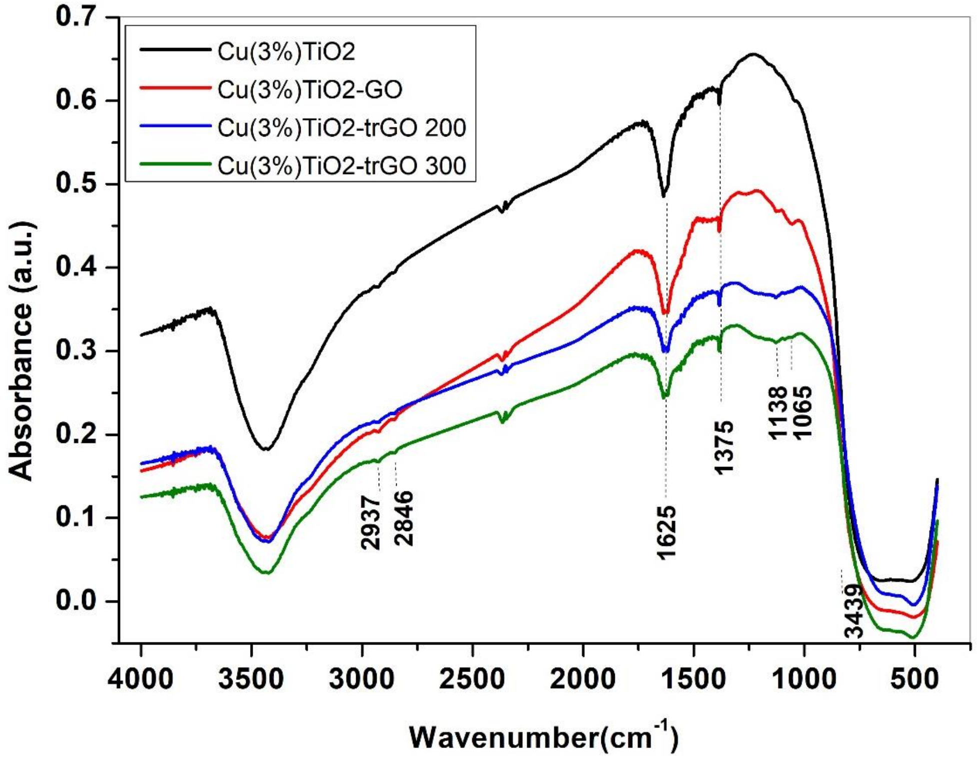

Figure S3. The FT-IR spectra of Cu(3%)-TiO_2-graphene composites comparatively with Cu(3%)-TiO_2.

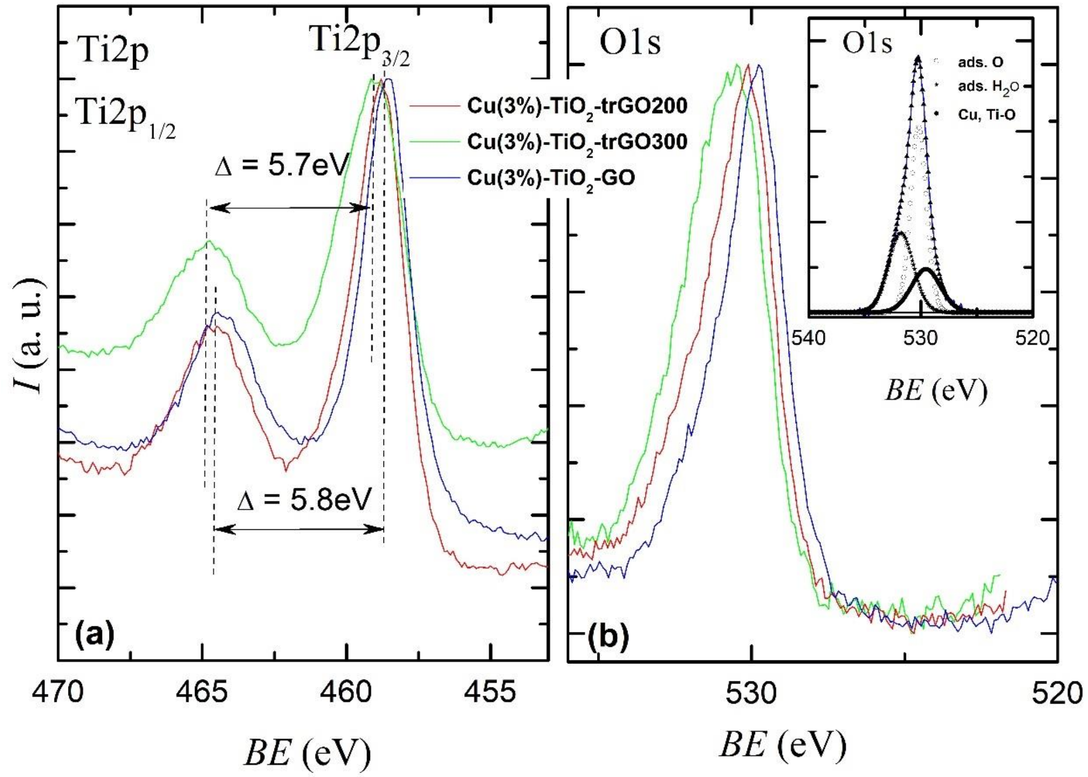

Figure S4. (a). Ti2p XPS for the investigated samples. Dashed line marks the spin orbit splitting of the Ti2p3/2 and Ti2p1/2 components. (b) O1s high resolution spectra for the investigated samples. Inset shows the deconvolution of O1s spectra for Cu(3%)-TiO_2-GO sample, as an exemplification for the identified chemical bonds at the surface of the sample.

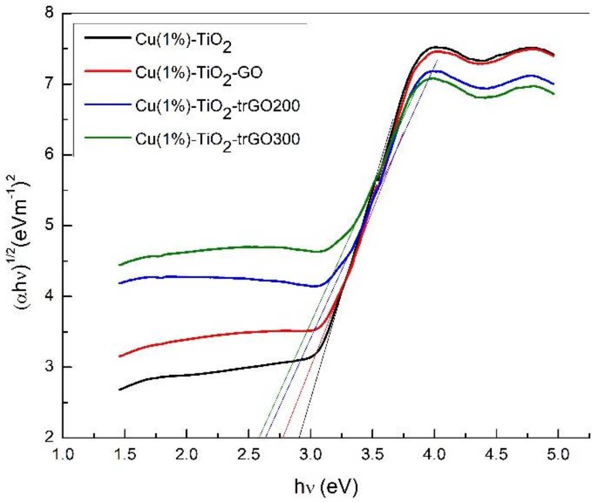

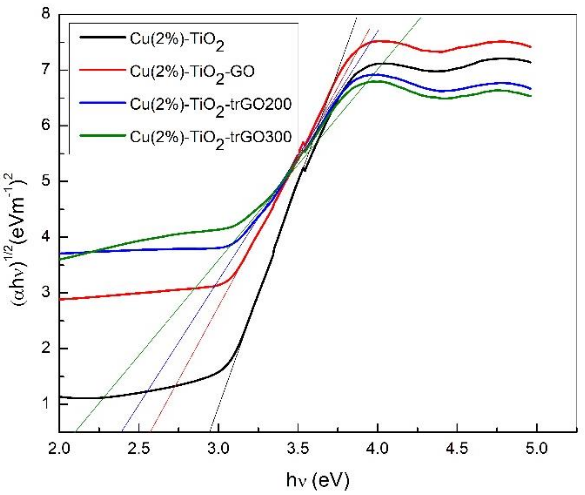

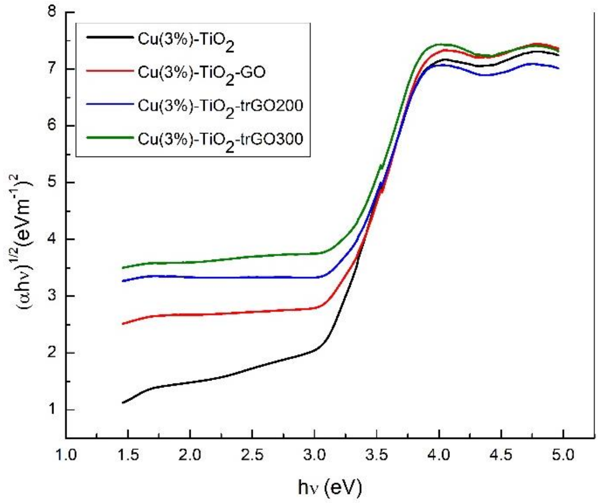

Figure S5. Tauc plots of all the analyzed samples (with 1%, 2% or 3% copper content).

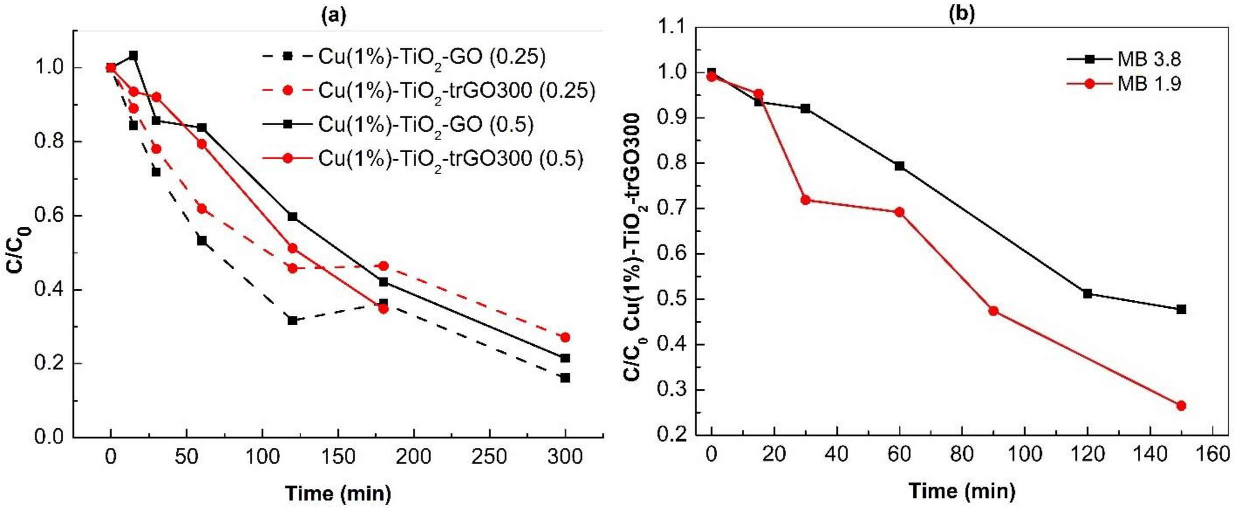

Figure S6. Residual ratio of MB (starting 5.5 mg/L) after UV-A irradiation (at 0.5 and 0.25 mg/mL photocatalyst dose) (a); the starting MB concentration of 3.8 and 1.9 mg/L (b).

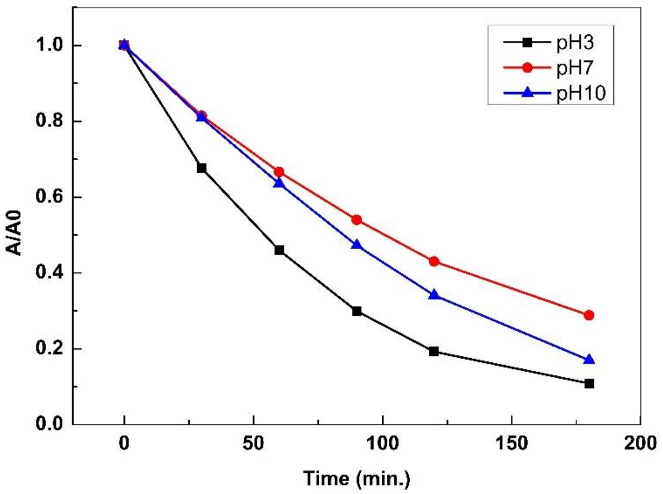

Figure S7. The pH influence over the residual ratio of MB (starting 5.5 mg/L) after UV-A irradiation in the presence of Cu(1%)-TiO_2-trGO300 photocatalyst.

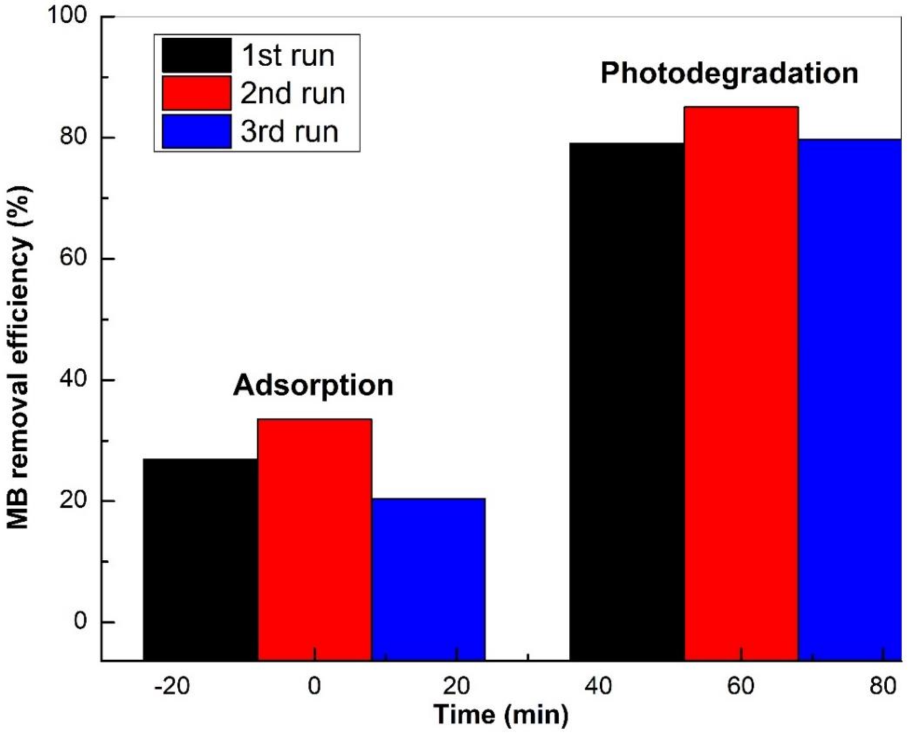

Figure S8. Recyclability study for Cu(1%)-TiO_2-trGO300, 5.5 mg/L MB solution pH = 7 (30 minute dark and 1 hour irradiation with UVA light)

Table S1. Elemental concentration at the surface of the investigated samples determined from XPS survey spectra of the analyzed samples.

Sample	Elemental concentration			
	**Cu**	**O**	**C**	**Ti**
**Cu(3%)-TiO_2-GO**	2	46.8	36.8	14.1
**Cu(3%)-TiO_2-trGO200**	4.2	45.8	34.8	15
**Cu(3%)-TiO_2-trGO300**	3.8	47.9	33.5	14.6

Table S2. Deconvolution components with the corresponding ratio determined from high resolution Cu2p spectra of the analyzed samples.

Sample		Cu2p		
		Cu_2O	CuO	Satellite
**Cu(3%)-TiO_2-GO**	Binding energy (eV)	932eV	933.3eV	942.3eV
	Relative concentration (%)	51.6%	31%	
**Cu(3%)-TiO_2-trGO200**	Binding energy (eV)	932.2eV	934eV	942eV
	Relative concentration (%)	20.6%	55.6%	
**Cu(3%)-TiO_2-trGO300**	Binding energy (eV)	932.5eV	934.4eV	943 eV
	Relative concentration (%)	22.7%	53.2%	

Table S3. Data obtained from the deconvolution of the C1s peaks of the investigated samples.

**Cu(3%)-TiO_2-GO**				
binding energy	284.2eV	284.6eV	285.8eV	288eV
Relative intensity	16%	26.2%	50.4%	7.4%
**Cu(3%)-TiO_2-trGO200**				
binding energy	284eV	284.7eV	285.8eV	287.8eV
Relative intensity	20.4%	37.5%	26.6%	15.8%
**Cu(3%)-TiO_2-trGO300**				
binding energy	284.1eV	285.1eV	286.3eV	288.5eV
Relative intensity	38%	30.7%	30.5%	7.9%

Table S4. The adsorption isotherm models (Langmuir, Freundlich and Temkin) parameters for MB on the studied photocatalysts

**Thermodynamic model**	**Parameters**	**Cu(1%)-TiO_2-GO**	**Cu(1%) trGO200**	**Cu(1%)-TiO_2-trGO300**	**Cu(3%)-TiO_2-GO**	**Cu(3%)-TiO_2-trGO200**	**Cu(3%)-TiO_2-trGO300**
**Langmuir**	q_{max} (mg/g)	-15.06	8.53	10.66	56.6	-264.5	-37.55

	K_L(L/mg)	-0.118	0.3193	0.2453	0.0628	-0.0135	-0.0891
	R_L	1.02	0.6622	0.7181	0.878	1.03	1.244
	R^2	**0.84955**	**0.9746**	**0.90445**	0.8512	0.7969	0.70818
**Freundlich**	K_F (mg/g (L/mg)$^{1/n}$	3.236	3.4972	3.4483	2.1017	2.0328	2.0895
	1/n	0.78405	0.8288	0.83586	0.94432	0.68845	0.6778
	R^2	0.7652	0.7446	0.70597	0.68694	**0.9166**	**0.81689**
